# An exploration of socio-economic and food characteristics of high trans fatty acid consumers in the Dutch and UK national surveys after voluntary product reformulation

**DOI:** 10.1080/16546628.2017.1412793

**Published:** 2017-12-05

**Authors:** H. L. Rippin, J. Hutchinson, M Ocke, J. Jewell, J. J. Breda, J. E. Cade

**Affiliations:** ^a^ Nutritional Epidemiology Group (NEG), School of Food Science and Nutrition, University of Leeds, Leeds, UK; ^b^ Centre for Nutrition, Prevention and Health Services, National Institute for Public Health and the Environment (RIVM), Bilthoven, The Netherlands; ^c^ Division of Noncommunicable Diseases and Promoting Health through the Life-Course, World Health Organization Regional Office for Europe, Copenhagen, Denmark

**Keywords:** Nutritional epidemiology group, World Health Organisation, trans fatty acids (TFA), industry product reformulation, voluntary TFA reduction, WHO TFA recommendations, socio-economic disadvantage, consumer characteristics, national dietary surveys

## Abstract

Trans fatty acids (TFA) increase the risk of mortality and chronic diseases. TFA intakes have fallen since reformulation, but may still be high in certain, vulnerable, groups. This paper investigates socio-economic and food consumption characteristics of high TFA consumers after voluntary reformulation in the Netherlands and UK. Post-reformulation data of adults aged 19–64 was analysed in two national surveys: the Dutch National Food Consumption Survey (DNFCS) collected 2007–2010 using 2*24hr recalls (N = 1933) and the UK National Diet and Nutrition Survey (NDNS) years 3&4 collected 2010/11 and 2011/12 using 4-day food diaries (N = 848). The socio-economic and food consumption characteristics of the top 10% and remaining 90% TFA consumers were compared. Means of continuous data were compared using t-tests and categorical data means using chi-squared tests. Multivariate logistic regression models indicated which socio-demographic variables were associated with high TFA consumption. In the Dutch analyses, women and those born outside the Netherlands were more likely to be top 10% TFA consumers than men and Dutch-born. In the UK unadjusted analyses there was no significant trend in socio-economic characteristics between high and lower TFA consumers, but there were regional differences in the multivariate logistic regression analyses. In the Netherlands, high TFA consumers were more likely to be consumers of cakes, buns & pastries; cream; and fried potato than the remaining 90%. Whereas in the UK, high TFA consumers were more likely to be consumers of lamb; cheese; and dairy desserts and lower crisps and savoury snack consumers. Some socio-demographic differences between high and lower TFA consumers were evident post-reformulation. High TFA consumers in the Dutch 2007–10 survey appeared more likely to obtain TFA from artificial sources than those in the UK survey. Further analyses using more up-to-date food composition databases may be needed.

## Introduction

Trans fatty acids (TFAs) are linked to all-cause mortality and various chronic diseases, most notably coronary heart disease (CHD) [1]. CHD causes around 10,200 deaths in the Netherlands [2] and approximately 73,000 deaths in the UK annually, making it the largest cause of mortality [3]. In addition, for every 2% total energy gained from TFAs there is a corresponding 23% increase in CHD incidence [4]. Industrial trans fatty acids (iTFA) are artificially produced in processed foods by hydrogenating vegetable or fish oils [5] and are associated with CHD mortality and total CHD [1]. Bakery products, spreads, packaged snack foods and deep-fried fast foods have been identified as major sources of iTFA [6]. The UK Low Income Diet and Nutrition Survey found unequal consumption of TFA by socio-economic status, with the most deprived groups having higher intakes of processed foods and takeaways [7]. Pearson-Stuttard et al. [8] suggest that reducing iTFA intake could substantially reduce health inequalities in CHD mortality. They estimated that a 1% reduction in TFA of daily energy intake would result in five times fewer deaths and six times more life years in the most deprived quintile than the most affluent.

The World Health Organisation (WHO) advises consuming <1% of total energy from TFAs [9]. To achieve this the WHO European food and nutrition action plan calls for a ‘virtual elimination’ of iTFAs [10]. Denmark was the first country to ban iTFA use by setting a maximum limit of 2g TFA per 100g total fat [11]. This has been effective in reducing iTFA content in foods and a decline in cardiovascular mortality has been directly attributed to the policy [11]. The ban was replicated in a number of European countries, including Austria, Iceland, Norway, Hungary and Switzerland [12]. Latvia plan to implement an iTFA ban by 2018, while Turkey and Georgia have introduced legal measures relating to labelling. Conversely, the UK and Netherlands have largely pursued voluntary iTFA reduction through product reformulation and advanced production techniques. However, voluntary approaches may have significant limitations; since they may not apply to the entire food supply chain, reformulation efforts may be uneven across product categories, and population subgroups could continue to consume high amounts even if the average population intake is at or below recommended levels [13]. The UK and Netherlands thus provide sound case studies for exploring potential advantages and drawbacks of voluntary approaches [14].

As part of public health iTFA reduction policies, the Dutch Task Force for the Improvement of the Fatty Acid Composition (Task Force verantwoorde vetzuursamenstelling: TFIFAC) launched in 2003. This initiative prompted manufacturers to reformulate and lower iTFA content in products, but was self-regulatory, so not monitored by government or an independent body [15]. In the UK, the Public Health Responsibility Deal (PHRD) iTFA reduction pledge [16] was introduced later, in 2011 in response to consumer health concerns. In this scheme businesses were permitted to reference actions undertaken prior to 2011, so new measures were not guaranteed across all PHRD signatories. However, their broadly common approach means that the impact of voluntary iTFA reduction in these countries can be explored and compared, with a view to advising other European countries. Further parallels are that both UK and Dutch TFA intake had been moderate compared to many other European nations; mean TFA intake in men was 1.3% total energy in the UK (1996) and 1.5% in the Netherlands (1992), compared to extremes of 2.1% in Iceland (1990) and 0.5% in Greece (1995) and Italy (1980–84). In addition, Sweden also employs a self-regulatory approach and had a moderate TFA intake [17].

Although the latest Dutch National Food Consumption Survey (DNFCS) [18] and UK National Diet and Nutrition Survey (NDNS) [19] reported that on average Dutch and UK adults meet the WHO and Dutch national limits of <1% total energy intake [9], these averages could still mask inequalities within certain groups, like those on low incomes [20]. For instance in Portugal, biscuits and pastry products typically imported and available in budget outlets have higher TFA levels than the majority of products in the country, which meet WHO guidelines [21].

The aim of this research is to analyse the Dutch and UK national nutrition surveys, which incorporate data gathered after voluntary product reformulation, to determine the characteristics of high compared to lower TFA consumers, and to determine whether similarities in Dutch and UK consumers exist, with particular reference to socially disadvantaged groups and consumption of food groups. The Dutch and UK examples could therefore advance understanding of the merits and limitations of voluntary TFA reduction in the context of minimising health inequalities.

## Methods

Two government-funded national surveys collected post-product reformulation were obtained and analysed for TFA content in relation to socio-economic and dietary characteristics [22,23].

### Dutch data

Data analysed from the Dutch National Food Consumption Survey (DNFCS) was collected March 2007-April 2010 [18] after industry product reformulation. The samples were drawn from consumer panels from Market Research GfK Panel Services, representative on age, gender, region, education and urbanisation. The DNFCS 2007–10 collected food data from individuals aged 7–69 using two 24-hour dietary recalls on two non-consecutive days by trained dietitians during home visits using the computer-based interview program EPIC–Soft, now called GloboDiet (©IARC) [18]. Portion sizes were given either by weight/volume or estimated using standard or household measures, or pictorial representations [18]. Demographic information was collected using age-specific general questionnaires [18].

TFA values in the DNFCS 2007–10 survey were based on values in the Dutch Food Composition Database, NEVO 2011 [24]. Nutrient data in NEVO 2011 originate from several sources in addition to the Dutch National Institute for Public Health and the Environment’s (RIVM) preferred chemical analyses from accredited laboratories. TFA values from the food industry collated by the Dutch task force (TFIFAC) informed the NEVO TFA values for potato products, bread, pastry, cakes and biscuits (excluding foods made with butter), (meat) snacks and salads, fats and margarines [15]. Other TFA sources include scientific publications, foreign food composition tables and derived nutrients from comparable foods [24].

### UK data

UK Data incorporating the reduced TFA content of reformulated products was analysed from years 3&4 (2010/2011 & 2011/2012) of the 2008–12 NDNS Rolling Programme (RP) [19]. Years 1&2 of the NDNS RP data were not included in the analyses because NDNS RP year 1 did not incorporate post-reformulation TFA compositions and year 2 data only incorporated some changes.

The UK NDNS 2008–12 RP collected data from individuals aged up to 64 using a consecutive four-day food diary; portion sizes were estimated using household measures and food packaging labels (Bates et al., 2014). The samples were drawn from UK Postcode Address Files, selected using multi-stage random probability sampling with postal sectors as the primary sampling units. Laboratory-analysed TFA levels in processed foods high in iTFAs and targeted for reformulation [25,26] were incorporated into the nutrient composition tables supporting years 3&4 (2010/2011 & 2011/2012) of the NDNS RP. These mostly popular and widely purchased products were gathered between 2008 and 2010 in the UK [25,26]. Sub-samples of the food products had been combined in equal weights to form a composite sample for analysis, with 5–16 sub-samples for each food sample category [26]. The UK Department of Health (DH) adopted the new TFA values where they were lower than in the existing composition tables; where they were equal or higher, existing values were retained.

### Statistical methods

The percentage of individuals who consumed more than the current WHO recommended limits on TFAs [9] i.e. more than or equal to 1% of their total energy from TFAs, was determined for Dutch and UK adults aged 19–64 using the intake averaged over 2 and 4 days, respectively. Due to low numbers in the UK post-reformulation surveys consuming above WHO recommended TFA limits (n = 22) and the potential distortion of total energy intake by high alcohol consumers, further analyses were conducted on the top 10% of TFA consumers as a percentage of food (rather than total) energy intake, defined as non-alcohol energy. These were then compared to the remaining 90% for adults aged 19–64, for both the Netherlands and the UK.

Characteristics of the top 10% TFA consumers were compared to lower TFA consumers using socio-demographic variables, which were the same or similar in the UK and Dutch surveys. These variables were: age, continuous and grouped (19-34y, 35-49y, 50-64y); gender; education (Dutch: High (University or higher vocational), medium (higher general secondary or Intermediate vocational), Lower (primary or lower vocational). UK: High (Degree), Medium (Qualifications below degree), Lower (No qualifications or in FT education); in employment (Yes/No); monthly income split into 5 groups (Dutch: net. UK: gross); region split into 4 groups (Dutch: 1) three largest cities in West Netherlands, 2) Rest of the West, 3) North, 4) East, South. UK: 1) London, East & South England, 2) North England, 3) Midlands, 4) Scotland, Wales & Northern Ireland); household size (number of people in household); Origin/Ethnicity (Dutch: native or non-Dutch native (born outside of the Netherlands). UK: (white or non-white)); smoking status (current, ex-regular, never regular); whether drinks alcohol (Yes/No). The means of continuous data were compared using t-tests and categorical data were compared using chi squared tests within country.

Food and nutrient intakes were also compared between the high and lower TFA groups. Where data was reasonably normally distributed, selected macro and micronutrient intake comparisons were undertaken by t-tests. Additionally, the percentage of consumers of selected food groups known to be high in TFA content were compared between the high and lower TFA intake groups using chi squared tests.

Multivariate logistic regression models for the Dutch and UK datasets were undertaken separately to determine which socio-demographic characteristics were independently associated with high TFA consumption. This incorporated age as a continuous variable; gender, qualifications as three categories (high, medium and low); income and region categorised as above; total number in the household and a binary ethnic origin variable.

Sensitivity analysis was conducted whereby all analyses were rerun after the identification and exclusion of under-reporters. Following the Oxford equations derived from Henry [27], survey height and weight data were used to generate Basal Metabolic Rate (BMR) and BMR:energy intake ratio variables. A general Physical Activity Level (PAL) of 1.55 was set to generate a low cut off via the Goldberg method [28]. For continuity, analyses were rerun after excluding under-reporters from the original top 10% group rather than generating a new top 10%.

All analyses were weighted using the survey weights provided to produce estimated results representative of the Dutch [29] and UK populations [30]. Statistical significance was set at p < 0.05. All analyses used Stata version 13 [31].

## Results

There were 1933 adults aged 19–64 in the Dutch and 848 in the UK analyses. In the DNFCS 2007–10, on average men and women aged 19–64 consumed 0.6% total energy (0.5% for men; 0.6% for women) and 0.6% food energy (0.6% for men and women) from TFAs. In the UK NDNS RP (Y3&4), adults consumed 0.5% total energy (0.5% for men and women) and 0.5% food energy from TFAs (0.5% for men and women). On average over the two survey days, 7.4% of Dutch adults consumed more TFAs than the current WHO recommended limits and Dutch national guidelines (5.7% males; 9.0% females, p = 0.01). In the UK NDNS RP 2.5% of adults consumed more than the current WHO recommended limits on TFAs (1.9% males; 3.0% females) over the four survey days. Consumers above the WHO recommended limits in the Netherlands had 1.3% (95%CI: 1.3, 1.4) of total energy intake from TFAs. This was similar to the UK, where 1.2% (95%CI: 1.1, 1.4) of total energy intake came from TFAs. Thus, in both surveys, TFA intake as a % total energy was more than twice as high in those not meeting WHO recommendations than those who did, who consumed 0.5% TFA from total energy (p < 0.001). Mean TFA intake in the Dutch survey was 3.5g (95%CI: 3.2, 3.7) in those not meeting the WHO recommendations – significantly higher (p < 0.001) than those meeting the recommendations (1.3g (95%CI: 1.3, 1.3)). In the UK this was 2.2g (95%CI: 1.9, 2.4) compared to 1.0g (95%CI: 1.0, 1.1) (p < 0.001).

For the top 10% TFA consumers in the Dutch survey, mean TFA intake was significantly higher than for the remaining 90% at 3.3g compared to 1.3g (Table 1). This is higher than the top 10% TFA consumers in the UK NDNS RP years 3&4 (1.9g compared to 0.9g).

**Table 1.** Socio-demographic characteristics of adult TFA consumers in the DNFCS 2007–10 and the UK NDNS 2010/11–2011/12.

Top 10% TFA adults (aged 19–64) as % food energy	DNFCS 2007–10				NDNS RP 2010/11 to 2011/12 (years 3 & 4)			
	Total unweighted Numbers N = 1933 (Weighted N = 2870)	Top 10% TFA as % food energy N = 188 (Weighted N = 286)	Remaining 90% N = 1745 (Weighted N = 2583)	p value	Total unweighted Numbers N = 848 (Weighted N = 1277)	Top 10% TFA as % food energy N = 88 (Weighted N = 130)	Remaining 90% N = 760 (Weighted N = 1147)	p value
Trans fatty acid intake mean g/day (sd)		3.3 (1.2)	1.3 (0.7)	<0.001		1.9 (0.6)	1.0 (0.5)	<0.001
Age mean (sd)		43.4 (12.8)	41.8 (12.5)	0.09		42.9 (13.1)	41.1 (12.8)	0.3
Age (years):								
19–34	810	29.9%	31.3%	0.1	245	29.0%	35.2%	0.5
35–49	542	32.0%	38.3%		330	36.9%	36.2%	
50–64	581	38.0%	30.5%		273	34.1%	28.6%	
Male	964	42.2%	50.8%	0.04	484	55.4%	48.6%	0.3
Female	969	57.8%	49.2%		364	44.6%	51.4%	
Higher education^a^								
High	421	28.5%	23.7%	0.3	237	32.1%	27.8%	0.1
Medium	892	39.4%	45.7%		471	44.3%	56.3%	
Low	620	32.1%	30.6%		138	23.6%	15.9%	
In employment								
Yes	1333	71.7%	71.8%	1.0	609	70.2%	73.7%	0.5
No	568	28.3%	28.3%		239	29.8%	26.3%	
Household income^b^								
Lowest income group	341	18.8%	16.6%	0.9	123	8.8%	13.4%	0.4
2	368	17.3%	19.0%		141	23.3%	18.4%	
3	486	23.7%	24.0%		173	28.8%	22.3%	
4	334	16.4%	17.7%		142	16.3%	22.2%	
Highest income group	404	23.8%	22.8%		150	22.7%	23.8%	
Region^c^								
1	301	18.2%	15.3%	0.4	201	23.8%	23.8%	0.2
2	563	26.6%	29.8%		148	24.2%	14.5%	
3	206	8.6%	11.0%		352	39.0%	45.5%	
4	408	25.0%	20.3%		147	13.0%	16.3%	
5	455	21.6%	23.6%					
Number in household (sd)		2.49 (1.18)	2.63 (1.33)	0.2		2.82 (1.17)	2.98 (1.35)	0.2
Ethnic group:								
White/Dutch native	1865	93.0%	97.1%	0.01	750	81.5%	85.6%	0.4
Non-white/non-Dutch native	68	7.0%	2.9%		98	18.5%	14.4%	
Smoking status:								
Current	513	19.4%	25.4%	0.2	204	33.0%	22.7%	0.1
Ex-regular	578	33.6%	31.8%		167	13.2%	19.1%	
Never-regular	841	47.1%	42.8%		477	53.8%	58.2%	
Whether drinks alcohol:								
No	599	32.2%	30.6%	0.7	287	37.6%	34.3%	0.6
Yes	1333	67.8%	69.4%		560	62.4%	65.7%	

^a^ Education for Dutch: High (University or higher vocational), medium (higher general secondary or Intermediate vocational), Lower (primary or lower vocational). For UK: High (Degree), Medium (Qualifications below degree), Lower (No qualifications or in FT education). ^b^Monthly net household income groupings for DNFCS 2007–10 are: less than EU1299, EU1300 to EU1899, EU1900 to EU2499, EU2500 to EU2899, EU2900 or more; Gross household income in last 12 months groupings for NDNS RP are: Less than £15,000, £15,000 to <20,000, £20,000 to <£35,000, £35,000 to <£50,000, £50,000 or more. ^c^ 1) Dutch regions: 1) three largest West Netherlands cities, 2) Rest of the West, 3) North, 4) East, 5) South. UK regions: London, East & South England, 2) North England, 3) Midlands, 4) Scotland, Wales & NI.

### Socio-demographic characteristics of the top 10% TFA consumers

In the Dutch analysis, there were significant differences relating to gender and ethnicity between the top 10% Dutch TFA consumers and remaining consumers (Table 1). Females were more likely to be top 10% consumers than males (Table 1). Non-Dutch natives were more likely to be in the top 10% TFA consumers and although not significant, in the UK analyses a higher proportion of non-white respondents were also in the top 10% TFA consumers (Table 1). In the unadjusted analyses there were no significant differences in other socio-demographic or socio-economic variables in either country, including age, education, employment status, income, geographic region or number of people in household. There were also no significant differences relating to smoking status or alcohol intake.

In the multivariate logistic regression analyses (Table 2) of the Dutch data, women were more likely than men to be top 10% consumers of TFAs (OR, 95%CI = 1.39, 0.99, 1.94; p = 0.05). Non-native individuals were nearly two and half times more likely to be top 10% consumers than Dutch native individuals (OR, 95%CI = 2.42, 1.16, 5.04). These non-natives were from Germany, France, Hungary, Poland, Romania, Brazil, Dutch Caribbean, Indonesia, Korea and Suriname. Aside from the second lowest income category in the UK data, which was more likely to feature in the top 10% TFA consumers, there were no significant differences related to income in either country. However, in the UK multivariate analysis, top 10% consumers were more likely to reside in the Midlands than in London, East and South England (OR, 95%CI = 2.30, 1.11, 4.78).

**Table 2.** Odds ratios (CI) of being in the top 10% adult TFA consumers by socio-demographic characteristic.

Top 10% TFA adults (aged 19–64) as % food energy	Mutually adjusted odds ratios (CI) of being in the top 10% as % food energy			
	DNFCS 2007–10 N = 1933 (Weighted N = 2870)	p value	Y3 & 4 NDNS RP N = 728 (Weighted N = 1068)	p value
Age	1.01 (0.99, 1.02)	0.1	1.01 (0.98, 1.04)	0.4
Male	1		1	
Female	1.39 (0.99 1.94)	0.05	0.83 (0.47, 1.44)	0.5
Qualification^a^				
High	1	0.2	1	0.2
Medium	0.74 (0.48, 1.13)	0.5	0.63 (0.33, 1.23)	0.4
Low	0.85 (0.53, 1.34)		1.55 (0.61, 3.95)	
Household income^b^				
Lowest income group	1	0.6	1	0.03
2	0.85 (0.49, 1.47)	1.0	2.46 (1.08, 5.61)	0.08
3	0.99 (0.58, 1.69)	1.0	2.25 (0.92, 5.52)	0.4
4	0.98 (0.56, 1.73)	0.9	1.49 (0.60, 3.69)	0.2
Highest income group	0.97 (0.56, 1.67)		1.76 (0.68, 4.59)	
Region^c^				
1	1	0.3	1	0.4
2	0.77 (0.47 1.27)	0.3	1.39 (0.65, 2.96)	0.03
3	0.69 (0.35, 1.36)	0.7	2.30 (1.11, 4.78)	0.7
4	1.09 (0.65, 1.82)	0.4	1.18 (0.49, 2.83)	
5	0.82 (0.49 1.37)			
Ethnic group^d^				
Native/White	1	0.02	1	0.6
Non-native/Non-white	2.42 (1.16, 5.04)		1.28 (0.52, 3.20)	
Number in household (sd)	0.95 (0.83, 1.08)	0.4	0.91 (0.77, 1.08)	0.3

^a^ Education for Dutch: High (University or higher vocational), medium (higher general secondary or Intermediate vocational), Lower (primary or lower vocational). For UK: High (Degree), Medium (Qualifications below degree), Lower (No qualifications or in FT education). ^b^ Household income groupings. For Dutch (net monthly): <EU1299, EU1300 to 1899, EU1900 to 2499, EU2500 to 2899, EU2900 and above. For the UK NDNS RP (Gross annual): less than £15,000, £15,000 to <20,000, £20,000 to <£35,000, £35,000 to <£50,000, £50,000 or more. ^c^ 1) Dutch regions: 1) three largest West Netherlands cities, 2) Rest of the West, 3) North, 4) East, 5) South. UK regions: London, East & South England, 2) North England, 3) Midlands, 4) Scotland, Wales & NI. ^d^ For Dutch: Native, Non-native. For UK: White, Non-white.

### Food and nutrition intake of the top 10% TFA consumers

In relation to foods and other nutrients consumed, the top 10% of TFA consumers in the UK NDNS (2010/11 to 2011/12) had higher fat and saturated fat and lower sugars and vitamin E intakes as %FE (Table 3). They were also more likely to be consumers of lamb, butter and cheese, and less likely to be consumers of crisps and savoury snacks and dairy desserts than the remaining 90%. In comparison, the Dutch top 10% TFA consumers also had significantly higher fat and saturated fat intake as %FE, and although the same pattern for these nutrients was observed, intake was higher than in the UK (Table 3). As in the UK, the top 10% Dutch TFA consumers consumed more butter than the remaining 90%, but there were no significant differences in lamb, cheese or crisps and savoury snack consumption. The top 10% Dutch TFA consumers did consume significantly more cream and buns, cakes and pastries (Table 3). The top 10% UK, but not Dutch TFA consumers also consumed less vitamin C and E than the remaining 90%.

**Table 3.** Diet-related characteristics of adult TFA consumers in the DNFCS 2007–10 and the UK NDNS 2010/11–2011/12.

Top 10% TFA adults (aged 19–64) as % food energy	DNFCS 2007–10				NDNS RP 2010/11 to 2011/12 (years 3 & 4)			
	Total Numbers N = 1933 (Weighted N = 2870)	Top 10% TFA as % food energy N = 188 (Weighted N = 286)	Remaining 90% N = 1745 (Weighted N = 2583)	p value	Total Numbers N = 848 (Weighted N = 1277)	Top 10% TFA as % food energy N = 88 (Weighted N = 130)	Remaining 90% N = 760 (Weighted N = 1147)	p value
Intake		Mean (sd)	Mean (sd)			Mean (sd)	Mean (sd)	
Trans fatty acid g		3.28 (1.20)	1.29 (0.68)	<0.001		1.93 (0.59)	0.95 (0.46)	<0.001
Trans fatty acid intake % of total energy		1.22 (0.33)	0.48 (0.19)	<0.001		0.95 (0.21)	0.46 (0.17)	<0.001
Trans fatty acid intake % of food energy		1.26 (0.32)	0.50 (0.19)	<0.001		1.00 (0.20)	0.49 (0.17)	<0.001
Total energy kcal		2444 (728)	2403 (817)	0.5		1853 (556)	1827 (556)	0.7
Food energy kcal		2365 (666)	2303 (744)	0.3		1762 (514)	1735 (519)	0.7
Fat intake %FE^a^		38.0 (5.8)	34.2 (6.6)	<0.001		39.0 (4.5)	33.8 (6.1)	<0.001
Saturated fat %FE^a^		15.5 (3.3)	12.7 (3.0)	<0.001		16.8 (3.2)	12.0 (2.9)	<0.001
Sugars^b^ %FE^a^		19.6 (6.6)	20.2 (7.2)	0.3		10.2 (5.7)	12.2 (6.5)	0.01
Vitamin C mg/1000kcal FE^a^		43.3 (29.9)	46.3 (31.9)	0.3		39.5 (21.9)	46.9 (37.1)	0.02
Vitamin D µg/1000kcal FE^a^		1.6 (0.8)	1.6 (1.0)	0.8		1.5 (1.2)	1.6 (1.1)	0.3
Vitamin E mg/1000kcal FE^a^		5.9 (2.3)	6.0 (2.4)	0.4		4.6 (1.3)	5.6 (2.4)	<0.001
Percentage consuming		% consumers	% consumers			% consumers	% consumers	
Beef ^c^	579	32.2%	31.0%	0.8	514	63.4%	58.2%	0.4
Lamb	38	3.4%	1.7%	0.1	123	34.3%	12.2%	<0.001
Burger	180	7.6%	9.4%	0.4	105	10.3%	14.4%	0.3
Sausages	890	44%	46.5%	0.5	319	33.5%	37.9%	0.5
Butter	379	30.1%	18.6%	<0.001	266	52.8%	28.5%	<0.001
Cream	327	33.9%	15.9%	<0.001	167	26.8%	19.5%	0.1
Whole milk	99	7.2%	5.0%	0.2	167	25.8%	18.7%	0.1
Ice cream	270	13.5%	14.5%	0.7	157	15.2%	19.8%	0.3
Crisps & savoury snack	768	36.9%	38.7%	0.6	477	33.3%	51.5%	0.003
Biscuits	1052	52.0%	55.4%	0.4	522	54.1%	61.1%	0.3
Buns, cakes, pastries	1078	84.6%	53.9%	<0.001	411	54.3%	49.1%	0.4
Cheese	1609	86.2%	82.8%	0.3	531	83.1%	61.7%	<0.001
Battered/coated/fried fish	54	2.6%	2.8%	0.5	186	23.4%	23.7%	1.0
Chips/fried potato etc.	729	40.4%	37.5%	0.5	528	58.6%	64.1%	0.4
Chocolate/confectionary	1300	68.6%	67.0%	0.7	349	42.2%	41.8%	1.0
Dairy desserts	549	33.8%	28.3%	0.1	335	25.8%	40.2%	0.02

T tests were used to determine p value for differences in intake, and chi squared tests for % consumers

^a^Diet only, does not include supplements/supplementation

^b^Dutch survey shows results for all mono and disaccharides, UK NDNS shows Non-milk extrinsic sugars.

^c^Beef, lamb and processed red meat consumption includes composite dishes in the NDNS RP.

### Sensitivity analysis

Sensitivity analysis to exclude under-reporters reduced the top 10% UK TFA consumer group by 50% (n = 44) compared to 12% for the Dutch (n = 165). The analysis showed a slight increase in the percentage consuming above the WHO and Dutch guidelines (7.7% Dutch and 2.6% UK adults) and also in the TFA intake of these over-consumers (3.6g for Dutch and 2.3g for UK adults). Intakes of total and food energy from TFAs either remained the same or showed marginal changes. Where changes occurred in socio-demographic and diet association, most became non-significant, including gender in the DNFCS and region, sugar and vitamin E intake and dairy dessert consumption in the NDNS. Exceptions included cream consumption becoming significant in the NDNS, age in the DNFCS and a strengthened association between non-Dutch natives and being in the top TFA consumer groups. The association remained between iTFA-containing buns, cakes and pastries in high Dutch TFA consumers and food groups characterised by ruminant TFAs (lamb, butter, cheese, cream) in high UK TFA consumers.

## Discussion

On average, the TFA consumed in the Dutch and UK nationally representative surveys was well below the WHO recommendations of <1% total energy, being 0.6% and 0.5% respectively. However, we found 7% of Dutch adults in the DNFCS 2007–10 consumed more than the WHO recommended limits; this group consumed 1.3% (3.5g) of their total energy from TFAs. Only 2.5% of UK adults in the NDNS RP (2010/11 to 2011/12) consumed over the WHO recommended limits; this group obtained 1.2% (2.2g) total energy from TFAs. Dutch women were more likely than men to feature in the top 10% consumers, possibly due to differences in dietary patterns. Those born outside the Netherlands were significantly more likely to be top 10% TFA consumers. There were no significant socio-economic associations, but in the UK multivariate analysis, top 10% consumers were more likely to reside in the Midlands, where incomes are generally lower than in London, East and South England [32]. The food consumption profile of the top 10% UK TFA consumers was predominantly ruminant-based (lamb, butter, cheese), but the Dutch higher TFA intake still featured both industrial and ruminant TFAs (butter and also cream, buns, cakes and pastries). Although the UK survey is more recent than the Dutch, voluntary measures to reduce TFA started much earlier in the Netherlands (2003) [15], than in the UK (2011) [16]. In addition to differences in the number of collection days used in the surveys, some of the differences in results between the countries may reflect differences in how recently and thoroughly the food composition tables underpinning the survey data have been updated, particularly regarding TFA.

We reported that a considerably larger proportion of adults (7%) consumed TFAs over the Dutch national and WHO recommended limits, than that in the Dutch DNFCS 2007–10 report, where recommended limits were exceeded by only 1–5% of the population depending on age group and gender [18]. Whilst we used the observed individual mean TFA intake over two collection days in our analyses of Dutch data, and over four collection days for the UK, habitual (usual) intake was estimated for the DNFCS 2007–10 report [18]. Collection of a limited number of days food intake can lead to considerable within-individual variation, which tends to widen intake distributions produced from observed individual mean intakes, resulting in overestimation of the more extreme percentiles [33]. The habitual intake distribution by age and gender used in the DNFCS 2007–10 report was estimated from the observed daily intake by correcting for the intra-individual (day-to-day) variation using SPADE (Statistical Program to Assess Dietary Exposure) [34]. In addition, the use of consumption data of two rather than four days for defining food consumer and high TFA intake groups may result in more misclassification for the Dutch data, than the UK.

It is difficult to establish how up-to-date were the food composition tables underpinning the TFA content of post-reformulated foods available during the survey periods. The UK uses industry updates [25,26] to inform composition data, which are ad hoc reviews rather than regular annual updates. Although in the Netherlands the TFIFAC monitored compositional TFA changes annually, and provided industry data for the Dutch Food Composition Database (NEVO) until 2010, the NEVO 2011 used for our Dutch analyses may not be as up-to-date as the composition database underpinning the UK analyses. Industry data does not make up the majority of TFA values in NEVO 2011 (contributing to only 10% of the TFA values for cakes and biscuits for instance), which indicates post-reformulation data may not have been fully incorporated. Nevertheless, retail margarines and frying and cooking fats, which were reformulated before 2006 [15], are likely to have been incorporated. This demonstrates the importance of updated information in food composition databases; Dutch and UK product reformulation is voluntary, so although guidelines such as the UK PHRD [16] may exist, no mandatory or uniform programme is guaranteed. Regular rather than ad hoc reviews of relevant food categories would be necessary to ensure national diet surveys consistently report accurate TFA intakes on which sound conclusions and effective policy can be based.

Pre- and post-reformulation TFA intake comparisons have been made between UK surveys (Hutchinson et al.) [35] and also for the Netherlands using modelling techniques with young adult survey data [15]. The analysis of both countries showed a decrease in average TFA consumption post-reformulation, and indicated total TFA intake comprised of fewer foods previously associated with iTFAs, and more ruminant products. However, our finding that a larger proportion of top 10% compared to lower Dutch TFA consumers consume cakes, buns and pastries contradicts the Temme finding that these foods contributed most to decreased TFA consumption. Nevertheless, iTFAs in these foods may have been reduced, with further reductions possible. Alternatively, TFA values in the Temme composition database may have used more-up-to-date TFIFAC information than that in NEVO 2011 (used in our analyses), though this may be unlikely.

Previous reviews have suggested that, globally, voluntary measures may be less effective than legislated limits in reducing TFAs in food products and inequalities in intake [13]. Furthermore, where voluntary reformulation has been pursued, there have been reported difficulties in ensuring the participation of a critical mass of manufacturers and retailers, especially small and medium-sized enterprises (SMEs), which dominate the food sector [14]. Thus, in some countries, a ban has been favoured to have maximum impact for all socio-economic groups and create a level playing field for companies. However, we found little evidence that higher TFA intake was associated with lower socio-economic status in both the Dutch and UK analyses. Although some associations were seen in relation to income in the UK multivariate analyses, those in the lowest income group were not more likely to be top 10% consumers than the highest income groups. This suggests there has been a sizeable response from industry across society. Indeed, the UK PHRD TFA voluntary pledge has over 90 signatories, including major large manufacturers and retailers (DH, 2014) and TFIFAC members in the Netherlands include major suppliers and customers of vegetable oils and fats in various sectors spanning a range of product categories [36]. TFIFAC reductions from 2003–2009 in the Netherlands include processed oils and fats, bakery products and raw materials, pre- and deep-fried potato products, and snacks. TFA reduction is no longer a priority target for the Dutch National Agreement to Improve Product Composition 2014–2020 [37]. However, we found non-native Dutch were more than twice as likely to be top 10% consumers as native Dutch, which may indicate that imported food into the Netherlands may contain more TFA, as found in Portugal [21], or that composition values are outdated. The latter may be more likely, as NEVO does not differentiate between imported and domestic foods, but takes an average. Therefore even substantial intakes of high-TFA imported products would be masked. In the UK analyses being a top 10% TFA consumer was not associated with ethnicity.

Direct comparisons were not made between Dutch and UK data due to the different methods used for data collection. The UK NDNS 2008–12 RP used a four-day consecutive food diary, with portion sizes estimated using household measures and food packaging labels [19]. The DNFCS 2007–10 collected food data using two 24-hour dietary recalls on two non-consecutive days, and gathered demographic information by questionnaire. Portion sizes were established using either estimates based on household measures and/or pictorial formats or recalled weight of food prepared and consumed [18]. In both surveys, the results are limited by self-reporting of intake, where no respondents weighed food intake [18,19]. There is evidence of under-reporting in both studies [38,39], by an estimated 30% in the UK study and 17% in the Dutch study [18]. This may explain why energy intake, in addition to TFA intake, was lower in the UK NDNS than the DNFCS 2007–10. Sensitivity analysis to exclude under-reporters resulted in reduced top TFA consumer groups, particularly in the UK, which shrank by 50%. As expected, the reduced UK top TFA consumer group had less power to uncover associations – this is evident in the sensitivity analysis results, where the majority of changes in association were to non-significance. There were no significant socio-economic or related associations after excluding under-reporters. Under-reporters were not excluded in the original analysis to preserve power and following European Food Safety Authority (EFSA) guidance [40]. However, the sensitivity analysis exposes under-reporting as an important limitation in drawing conclusions based on this survey data.

The TFA values used in the UK NDNS survey is an average of a small variety of popular foods from large manufacturers and retailers; this average could mask important TFA differences between foods regularly purchased by different groups in society. For instance, fewer large retailers and more SMEs in deprived areas may mean lower income groups consume more non-reformulated, low budget foods potentially higher in TFA than values in the NDNS obtained from average composite samples. Similarly for the Netherlands, the TFIFAC data in the NEVO tables is equally unlikely to include information on specific low budget foods from SMEs. In addition, unpackaged foods from local independent outlets may be prepared using fats procured business-to-business, containing an unknown quantity of TFAs e.g. pastry shortening [21] which are unlikely to be part of national voluntary reformulation efforts. Further research in this area is needed to explore whether low budget or niche brand/international foods from SMEs have higher TFA content than more popular products underpinning nutrient databanks in both countries.

## Conclusion

According to the national dietary surveys of the Netherlands and the UK, both populations have low average TFA intakes, a state contributed to by successful voluntary national reduction programmes. Dutch people in the top 10% TFA consumers were more likely to be women and non-native. In the UK, the top 10% consumers were more likely to reside in the Midlands and had a more ruminant based TFA profile, whereas the Dutch appeared to obtain TFA from artificial as well as ruminant sources. It is possible that TFA intakes are underestimated in both countries due to under-reporting and the nature of food composition databases; inequalities in TFA consumption of certain vulnerable groups cannot be ruled out. This study demonstrates the need to investigate and evaluate the merit and impact of different iTFA removal policies, including voluntary reformulation, to ensure that no population groups have increased exposure via type or combination of food consumed. Regardless of policy approach, disaggregated consumption and updated food composition data should be used to determine whether high-TFA (including imported) products remain on the market.
